# A new perspective on tumor progression: Evolution via selection for function

**DOI:** 10.1093/emph/eoae021

**Published:** 2024-09-19

**Authors:** Frédéric Thomas, James DeGregori, Andriy Marusyk, Antoine M Dujon, Beata Ujvari, Jean-Pascal Capp, Robert Gatenby, Aurora M Nedelcu

**Affiliations:** CREEC/CANECEV, MIVEGEC (CREES) Department, University of Montpellier, CNRS, IRD, Montpellier, France; Department of Biochemistry and Molecular Genetics, University of Colorado Anschutz Medical Campus, Aurora, CO, USA; Department of Cancer Physiology, H Lee Moffitt Cancer Center and Research Institute, Tampa, FL, USA; CREEC/CANECEV, MIVEGEC (CREES) Department, University of Montpellier, CNRS, IRD, Montpellier, France; School of Life and Environmental Sciences, Deakin University, Geelong, Centre for Integrative Ecology, Waurn Ponds, VIC 3216, Australia; School of Life and Environmental Sciences, Deakin University, Geelong, Centre for Integrative Ecology, Waurn Ponds, VIC 3216, Australia; Toulouse Biotechnology Institute, University of Toulouse, INSA, CNRS, INRAE, Toulouse, France; Department of Cancer Physiology, H Lee Moffitt Cancer Center and Research Institute, Tampa, FL, USA; Department of Biology, University of New Brunswick, Fredericton, New Brunswick, Canada

**Keywords:** perspective, tumors, progression, evolution, selection, function, group phenotypic composition

## Abstract

Tumorigenesis is commonly attributed to Darwinian processes involving natural selection among cells and groups of cells. However, progressing tumors are those that also achieve an appropriate group phenotypic composition (GPC). Yet, the selective processes acting on tumor GPCs are distinct from that associated with classical Darwinian evolution (i.e. natural selection based on differential reproductive success) as tumors are not genuine evolutionary individuals and do not exhibit heritable variation in fitness. This complex evolutionary scenario is analogous to the recently proposed concept of ‘selection for function’ invoked for the evolution of both living and non-living systems. Therefore, we argue that it is inaccurate to assert that Darwinian processes alone account for all the aspects characterizing tumorigenesis and cancer progression; rather, by producing the genetic and phenotypic diversity required for creating novel GPCs, these processes fuel the evolutionary success of tumors that is dependent on selection for function at the tumor level.

## THE PREMISE

Following the pioneering work of Cairns [1] and Nowell [2], tumorigenesis has been generally viewed as underpinned by a classical Darwinian evolutionary process (i.e. somatic evolution) primarily governed by natural selection among mutant clones differing in fitness (i.e. survival and reproduction), starting from the emergence of precancerous lesions to deadly metastatic cancers. Selection, for example, can favor clones possessing mutations that directly enhance their proliferation capacity or survival, being capable of inducing angiogenesis, evading the immune system and therapies or being the most effective disseminators (leading to metastases) [3]. In addition to individual cells, selective processes can also affect groups of cells within a tumor. That is, by engaging in inter-clonal cooperative interactions, a group may collectively have increased fitness benefits relative to adjacent groups in, for example, their ability to engage in angiogenesis, invasion or protection from immune attacks [4]. Consequently, while a primary tumor may originate from a single cell or a small homogeneous cluster, as it expands and accumulates mutations and epigenetic alterations, it becomes a heterogeneous mix of distinct clones/lineages characterized by novel spatial arrangements and various interactions (competitive or cooperative), leading to changes in its phenotypic composition. However, at present, tumor growth is assumed to simply be the result of individual cell or group-cell phenomena. This view likely reflects our ability to apply mathematical and population genetics models to such cell- or group-level processes. Yet, these phenomena on their own cannot fully explain the evolutionary trajectory of a tumor; namely, why some tumors progress while others regress or remain stable for a long time. We propose that the Darwinian processes classically evoked to explain tumorigenesis are not sufficient to account for it entirely; they represent only a part of the explanation. We suggest here that another evolutionary dynamics that takes into account the specificities of tumor heterogeneity in terms of their phenotypic composition is essential for a comprehensive understanding of tumorigenesis. We hope that increasingly rich data on the spatial and genetic architecture of tumors will allow a better characterization of tumor phenotypic composition and its dynamics, which could translate into predictive models with therapeutic relevance.

## TUMOR HETEROGENEITY, SELECTION AND GROUP PHENOTYPIC COMPOSITION

**Figure 1.**
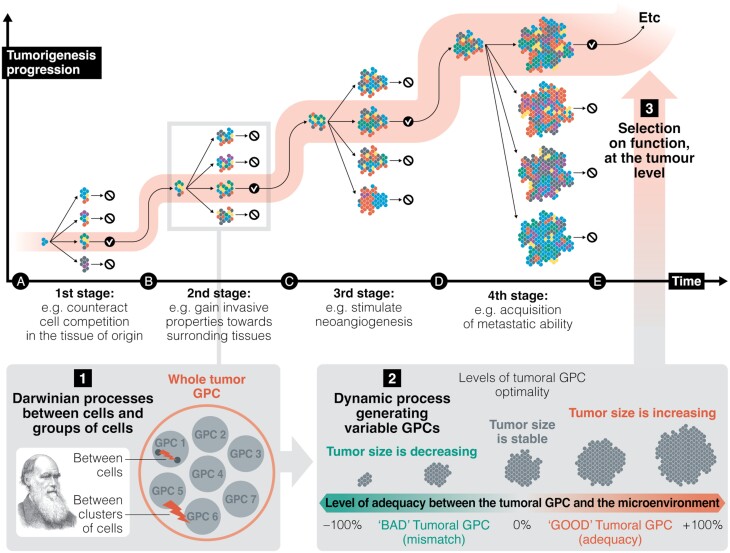
Nested selective processes are responsible for the long-term evolutionary success of a tumor. The example above represents progression toward a malignant tumor, but as indicated in the main text, this is not necessarily the only possible scenario. (A) The first stages of tumorigenesis are driven by Darwinian processes among individual cells and clusters of cells. (B) These Darwinian processes also constantly and dynamically generate variable GPCs in the tumor. (C) The generation of variable GPCs propels the tumor into a second dynamic characterized by selection for function, in which tumor growth occurs when there is alignment between the tumor’s functionality (as defined by Wong *et al.* [7]) induced by the GPC and the current microenvironment. Unraveling these two evolutionary dynamics and the mechanisms that drive them is essential for fully understanding tumorigenesis. Figure adapted from Capp *et al.* [5].

Capp *et al.* [5] recently transposed to tumors the ecological concept of ‘group phenotypic composition’ (hereafter GPC) (see Ref. [6], as well as Fig. 1) to describe the spatio-functional distribution of the intra-tumoral heterogeneity throughout tumor progression, in relationship with the changing (i.e. context-dependent) tumor microenvironment. The whole tumor GPC displays a nested and dynamic structure in which each functional cluster within the tumor has its own changing GPC. GPCs represent dynamic networks with elements of both cell- and cluster-level selection. That is, within a cluster, selection on individual cells may result in cooperating or competing dynamics, which will affect the fate of the cluster. For example, a group of cooperating cells promoting angiogenesis might have survival benefits relative to non-cooperative clusters. However, a cell within the cluster could ‘cheat’ by not producing pro-angiogenic factors. The cheater benefits from the group activity (i.e. angiogenesis) but does not bear the cost, resulting in its increased proliferation and a change in the GPC of the cluster that will ultimately affect the overall tumor composition and its progression potential. Thus, in addition to contributing to the overall tumorigenesis, the GPC model predicts a level of interaction and communication across small spatial scales that are not currently recognized [5].

Tumors that exhibit an oncogenic GPC (at both the cluster and tumor levels) in relation to their microenvironment at each time/stage are the ones that thrive and progress; that is, they are ‘selected’. However, this is an atypical selective process (i.e. distinct from the classical biological natural selection process) since it is inappropriate to talk of differential reproductive success or heritable variation in fitness for tumors. Rather, tumors are analogous to natural populations (especially in those in which members can specialize in various activities and both cooperative and competitive interactions can occur—such as, for instance, human populations) that evolve because of selection among its members, but whose evolutionary success/stability or extinction is dependent on (i) the individual fitness of its members, (ii) possible interactions among individuals and (iii) the ability to change their composition in response to the environment. In this view, a tumor is a complex and dynamic society of cells, whose continuous success is dependent on adjusting its phenotypic composition, which, in turn, reflects the various fitness levels of individual cells and the type of interactions cells are involved in. Notably, although GPCs characterize the tumor level, they are the result of evolutionary processes and outcomes at the cell and cell-group levels. In that sense, although GPCs can benefit the tumor they are not true adaptations at the tumor level; rather, they can be considered cross-level byproducts [8] that can nevertheless affect the fate of the tumor. In addition, the progression of tumors, as with any evolutionary process, will be influenced by the status of the individual harboring the tumor because both processes at the cell level—mutation and selection, will be influenced/affected by the genetic makeup (e.g. germline mutations that can increase mutation load) and the lifestyle (e.g. diet, smoking, exposure to mutagens) of the individual.

## TUMOR PROGRESSION: A PERSPECTIVE OF EVOLUTION VIA SELECTION FOR FUNCTION

The prevailing evolutionary view of cancer prioritizes classical Darwinian processes among cells and cell groups (i.e. somatic evolution), and regards the tumor’s growth and progression as byproducts of these cell-level processes resulting in some clones outcompeting others. Here, we aim to shift this perspective by suggesting that these Darwinian processes between cells and groups of cells serve as a means that allows a selectable tumor progression process, which revolves around possessing the appropriate tumor-promoting GPC for a specific context. This strategy is partially comparable to bet-hedging [9] but unlike classical bet-hedging that generates fixed biological entities (e.g. seeds with different germination dates in desert plants, e.g Ref. [10]), it is characterized by its dynamism and adaptability over time, thanks to the continuous involvement of underlying Darwinian processes at the cell level. Hence, while tumorigenesis is first initiated by cell-level processes, these processes quickly transition into a means of driving another evolutionary dynamic, at the tumor (i.e. as a complex system) level. This dynamic produces different GPCs, which can variably promote tumor persistence (or loss), tumor growth (or regression), and/or a more malignant (or benign) phenotype—depending on context (tumor and systemic microenvironments and accumulated changes in and among tumor cells), with variable effects on the host, ranging from largely benign to life-threatening. To the best of our knowledge, this distinction between the importance of the various evolutionary processes involved in tumorigenesis and their respective roles in selectable outcomes at the tumor level has not been considered before.

Our focus on the dynamics of GPC (and its multiple configurations) as well as selection of successful GPCs is consistent with the view of Wong *et al.* [7] who recently proposed a new evolutionary law that acknowledges evolution as a common characteristic of all complex systems in the natural world. These systems are composed of numerous different components, such as atoms, molecules or cells, which can be arranged in various ways and are subject to natural processes that generate many different configurations. Only a small fraction of these configurations survives through a process they coined ‘selection for function’. When a new configuration works well and enhances functionality, evolution occurs. This Law of Increasing Functional Information applies to a variety of systems, from stars to minerals to biology. The implications of the law include a better understanding of a system’s capacity to continue evolving, insights into how to artificially influence the rate of evolution, and a deeper understanding of the generative forces behind complexity in the universe. Overall, this new law suggests that evolution is a common phenomenon in a wide range of complex systems, and it relies on selection for function. The authors identified three types of function in nature: stability, dynamics with continuous energy input, and novelty, which is the tendency of evolving systems to explore new configurations that may lead to surprising new behaviors or characteristics.

Since tumors are complex and heterogeneous systems of cells that do not fulfill the requirements for Darwinian evolutionary individuals at the tumor level (i.e. heritable variation in fitness), we suggest that they follow a classical Darwinian framework at the cellular and cell cluster levels but undergo selection for function at the tumor level (Fig. 1). Tumors display the three attributes that Wong *et al.* [7] identified as common to all evolving systems (from minerals to stars, atmosphere and life): ‘1) they form from numerous components that have the potential to adopt combinatorially vast numbers of different configurations; 2) processes exist that generate numerous different configurations; and 3) configurations are preferentially selected based on function’.

Indeed, due to both high mutational and epigenetic changes that generate numerous different cell phenotypes with various fitness levels, vast numbers of different configurations can be adopted. In other words, evolving tumors are those which, by chance, create over time new configurations/GPCs (i.e. through cell-level processes involving natural selection and drift) that will be preferentially and continuously selected/maintained because they result in continuous improvements in tumor functionality in response to changes in the microenvironment. Tumor functionality can include either of the three types of functions envisioned by Wong *et al.* [7]: static persistence, dynamic persistence, and novelty generation. For instance, the ability of a tumor to maintain its size (through adaptively changing its GPC) in response to immune attacks and microenvironmental changes can be viewed as a selection for ‘static persistence’, whereas its growth in response to various intra-tumor and microenvironmental changes (e.g. through increased plasticity, inducing angiogenesis) might involve selection for ‘dynamic persistence’. Similarly, the acquisition of the ability to invade and migrate (i.e. a novel capability) can be considered the result of selection for ‘novelty generation’.

According to the law of increasing functional information proposed by Wong *et al.* [7], the system (i.e. tumor) will evolve if ‘many different configurations of the system undergo selection for one or more functions’. We posit that only tumors that evolve via selection for function at every stage will be evolutionary successful (i.e. do not go extinct) in the long run (see Fig. 1). The mechanism underlying this selective process is the generation of suitable GPCs that increase tumor functionality at each stage and in each context. Although this process might be viewed as a ‘survivor bias’ (i.e. tumors that fail to acquire the right mutations are typically not detected), we propose that cancer progression represents an example of a selection for function that is enabled by the ability to evolve tumorigenic GPCs (at both the cluster and tumor level).

Finally, we suggest that the new evolutionary dynamics proposed in this paper could also apply to non-cancerous processes in some cases. As individuals age, it is indeed common to observe the development of cellular growths that are not necessarily cancerous. These growths, often referred to as benign tumors or lesions, can manifest in various forms such as polyps, cysts, or lipomas [11]. Unlike malignant tumors, these formations do not spread to other parts of the body and generally do not pose a life-threatening risk. However, we propose that the persistence and increasing size and morphological complexity of some of these growths could also be attributed to selection for function, similar to malignant tumors. Further work is necessary to address these aspects, as well as to elucidate potential connections with the increased genetic mosaicism that has been described in normal tissues during aging (see Ref. [12] and references therein).

## THE SELECTION FOR FUNCTION IN A CONSTANTLY CHANGING ENVIRONMENT IS ACHIEVABLE THROUGH A DYNAMIC PROCESS THAT GENERATES DIVERSITY

Although often referred to as ‘tumor development’, tumor progression is not characterized by a predetermined roadmap [13] (see however Ref. [14]). Also, in contrast to living organisms that reproduce and pass down their genetic information, tumors emerge within their hosts and fade with them, precluding any avenue for transmission of new true adaptations at either the cell or tumor level (with the notable exception of transmissible cancers [15]). Consequently, tumorigenesis represents a perpetual cycle of trial and error, where the evolutionary strategy must pivot on processes producing permanent diversity within the current host. Traditional processes that generate diversity, such as bet-hedging, most often result in a fixed array of entities and are inadequate in this context. Tumor progression demands a dynamic system capable of continuously adjusting to the ever-shifting microenvironment. Because flexibility and adaptability are imperative, a dynamic process becomes paramount for allowing the tumor’s long-term maintenance and progression. One way to implement such a dynamic process involves repurposing the very Darwinian processes (competition or cooperation between cells and/or groups of cells) that initially triggered tumor development into creating a diversity of configurations. Thus, cell-level processes serve as a wellspring of proposals for various GPCs. The interplay of selection mechanisms amid the diverse GPCs created in this way would drive tumor evolution through selection for function (Fig. 1). Since changes in GPCs are driven by changes in the microenvironment, it follows that microenvironments that change rapidly and impose new selective pressures might favor tumors that are fast evolving (and the opposite) in terms of functionality. An equally interesting research direction to consider would be the idea that selection for function leads to GPCs that help shape a tumor microenvironment favorable to the tumor and its progression. Understanding these processes can allow us to predict tumor progression or direct its evolutionary strategy towards a less successful outcome.

## THERAPEUTIC IMPLICATIONS

From a conceptual point of view, our proposal involving an amended view on evolutionary processes affecting tumorigenesis (Fig. 1) has important therapeutic implications. A common goal of cancer therapy is eradication of all members of the tumor cell population. However, it is well established that attempts to fully eradicate a tumor carries the risk of selecting resistant clones that will be difficult to combat later. In addition, the GPC model suggests that current cancer treatments can also impose selection on groups of cells which may, therefore, collectively develop resistance mechanisms, and alter the overall tumor GPC and its evolutionary success. That is, such treatments can select for more ‘functional’ tumors ‘adapted’ to the new microenvironment. Interestingly, surviving cells following neo-adjuvant therapy have been often found clustered in tumors (e.g. Ref. [16]).

As a corollary, therapies that do not focus on eradicating the tumor are less likely to induce resistance, adaptive therapy being an emblematic example [17]. For instance, in metastatic castration-resistant prostate cancer, where curative treatments are currently not feasible, strategically administering currently available drugs based on principles of evolutionary ecology (such as the cost of resistance) has demonstrated the potential to establish an evolutionary stable therapy, as predicted by mathematical models [18]. This approach aims to maintain a stable population of polymorphic cells that are either resistant or sensitive to treatment, thereby ensuring sustained treatment efficacy and improving patient survival. Significant progress has been made in understanding the conditions necessary for optimal tumor control, which can stabilize tumors within a GPC range that enhances their responsiveness to treatments [19] (for specific examples see, for instance [20]).

In the framework presented here, the aim is to modify selection on the basis of tumor function, either directly (by altering its GPC qualitatively or quantitatively) or by creating conditions that favor tumors whose GPC do not facilitate progression toward aggressive cancers. It is conceivable in a therapeutic context to establish a rotation system that would induce the successive selection of GPCs within a loop (e.g. three therapies 1, 2, 3, 1, 2, 3, etc.), which always prevents evolution towards a GPC that can drive progression into aggressive tumors. This amounts to using selection for function to push the tumor toward an evolutionary trajectory that is not detrimental to the host and could improve prognosis.

Overall, we suggest that future efforts should be directed towards investigating tumor composition (in terms of its GPCs) as an alternative/additional approach to current studies on the presence of specific therapeutically targetable mutations. Once our understanding of what type(s) of GPC promote tumor progression (and the associated microenvironmental changes), we might be able to develop a series of markers or scores defining tumors that have the potential to reach the configuration (i.e. GPC) associated with progression (i.e. that could be ‘selected’ for aggressivity). Similarly, once we understand the evolutionary dynamics at the cell level that result in oncogenic GPCs, we can develop and apply therapeutic strategies to alter the GPC towards less aggressive tumors through directed interventions that target a specific clone or a specific aspect of the microenvironment.

## CONCLUDING REMARKS

Early tumorigenesis involves classical Darwinian processes affecting individual cells and cell groups. However, as a tumor progresses, these Darwinian processes at the cell and cell-group levels also randomly generate various GPCs that might or might not be in alignment with the microenvironment. Tumors with the right GPC for the corresponding microenvironment will be favored and progress—that is, are selected (i.e. are evolutionarily successful). This second selective process (Fig. 1) resembles the more universal concept of evolution through selection for function, which can be applied to complex systems that do not fit the classic Darwinian requirements for evolution, such as heritable variation in fitness, competition and differential reproduction. This perspective on tumorigenesis goes beyond the conventional notion that the selection on individual cells is the sole determining factor, from the early precancerous lesions to the most aggressive metastatic stages. Instead, it offers a comprehensive view that accommodates the complexity of tumors and evolutionary dynamics that govern tumor progression to explain the success of some but not all tumors. Natural biological selection among individual cells and cell groups, which is originally the engine of tumor initiation, later serves as a tool to generate various configurations (i.e. GPCs) suitable for various changing and challenging environments, which is the key to selection for functional optimization and tumor progression. This nuanced understanding of tumorigenesis, summarized in Fig. 1, paves the way for a deeper understanding of tumor heterogeneity and its significance for cancer progression, potentially leading to more targeted therapeutic interventions and a refined approach to cancer prevention and treatment.
